# IL-32 promotes the occurrence of atopic dermatitis by activating the JAK1/microRNA-155 axis

**DOI:** 10.1186/s12967-022-03375-x

**Published:** 2022-05-11

**Authors:** Jing Chang, Bin Zhou, Zhu Wei, Yongqi Luo

**Affiliations:** Department of Dermatology, Hunan Children’s Hospital, No. 68 Ziyuan Road, Changsha, 410007 People’s Republic of China

**Keywords:** Interleukin-32, Janus-activated kinase-1, microRNA-155, Atopic dermatitis, Human immortalized keratinocytes, Skin reconstruction model, Phosphorylation, Inflammation

## Abstract

**Background:**

This study aims to explore the mechanism of interleukin-32 (IL-32) affecting atopic dermatitis (AD) through the Janus-activated kinase-1 (JAK1)/microRNA-155 (miR-155) axis.

**Methods:**

In this study, skin tissue samples and blood samples from normal subjects and patients with AD, human immortalized keratinocytes (HaCaT), and PA-induced mouse models of AD were selected for expression determination of IL-32, JAK1 and miR-155. The interaction among IL-32, JAK1 and miR-155 was identified with their roles in AD analyzed through loss- and gain-of-function assays.

**Results:**

Elevated IL-32 was detected in AD tissues and blood samples and promoted the occurrence of AD. IL-32 upregulated JAK1 expression and phosphorylation of its downstream genes, thus activating the JAK signaling pathway. JAK1 promoted the expression of miR-155. IL-32/JAK1/miR-155 axis promoted inflammation in the AD skin reconstruction model. In vivo experiments further confirmed that IL-32 promoted AD development by activating the JAK1/miR-155 axis.

**Conclusion:**

The present study underlined that IL-32 promoted the occurrence of AD by promoting JAK1 expression to upregulate miR-155 expression.

**Supplementary Information:**

The online version contains supplementary material available at 10.1186/s12967-022-03375-x.

## Background

Atopic dermatitis (AD) is a chronic inflammatory disorder with increasing prevalence worldwide [1]. AD is prone to relapse and is characterized by serious pruritus that impacts the quality of patients’ life [2]. The pathophysiology of AD is highly complex, which involves skin barrier dysfunction as well as abnormal type 2 inflammation or immune responses [3]. The standard medical treatment for AD is focused on the symptomatic relief through control of skin inflammation using topical corticosteroids and/or calcineurin inhibitors [4]. Of note, the complicated pathogenesis of this skin disease still needs further exploration and there is still a lack of effective treatment options [5]. Against this backdrop, it is of significance to identify novel targets responsible for the occurrence and development of AD in hope of seeking novel treatment direction.

Interleukin-32 (IL-32) is identified as a type of pro-inflammatory cytokine that is generated by T lymphocytes, natural killer cells, monocytes, as well as epithelial cells [6]. It has been highlighted that IL-32 is responsible for the pathophysiology of AD at an early stage and elevated IL-32 is detected in the lesional skin and serum of patients with AD [7]. Moreover, the serum level of IL-32 in patients with AD is related to the disease severity [8]. Of note, IL-32 contributes to activation of Janus-activated kinase-1 (JAK1) to promote immune-mediated inflammation of rheumatoid arthritis [9]. Importantly, the use of delgocitinib that is capable of inhibiting the JAK family including JAK1 has been approved in Japan as a treatment regimen for AD [10]. Intriguingly, the important role of microRNAs (miRs) in AD has been unveiled [11]. As previously reported, upregulation of miR-155 has been observed in patients with AD in comparison with the healthy controls [12]. Strikingly, the interaction between miR-155 and JAK1 has been reported in T cells, which regulates the inflammatory response [13]. Given all the above evidence, we hypothesized that IL-32 may regulate the development of AD, with the participation of the JAK1/miR-155 axis.

## Materials and methods

### Ethical approval

The current study was approved by the Ethics Committee of Hunan Children’s Hospital and performed in strict accordance with the Declaration of Helsinki. All participants in this study signed informed consent documentation before sample collection. Animal experiments were approved by the Animal Ethics Committee of Hunan Children’s Hospital and strictly performed according to the Guide for the Care and Use of Laboratory Animals published by the US National Institutes of Health. Due efforts were made to limit animals’ pain.

### Clinical sample collection

Normal subjects (n = 50) and patients with AD (n = 90) who were treated in the dermatology department of Hunan Children’s Hospital from June 2018 to October 2019 were selected for collection of blood samples and tissue samples (n = 10 for each group). Patients were included if they (1) aged between 6 and 12 years (including the boundary value) at the time of signing informed consent, regardless of gender; (2) had moderate or severe AD; (3) had condition not fully controlled by topical prescription drugs or not suitable for topical drug treatment. Patients were excluded if they had (1) cardiovascular, neurological, renal, liver, digestive tract, urogenital system, psychiatric, nervous system, musculoskeletal, skin, sensory, immune, endocrine (including uncontrolled diabetes or thyroid disease) or uncontrolled hematological abnormalities; (2) other active skin diseases (such as psoriasis or lupus erythematosus) or skin infections (bacteria, fungi or viruses) that might affect the evaluation of AD; (3) severe concomitant diseases (such as unstable chronic asthma) that may receive systemic hormone therapy or other interventions or need active and frequent monitoring.

### Cell culture and transformation

Human immortalized keratinocytes (HaCaT) cell line (Cell Resource Center, Chinese Academy of Medical Sciences, 1101HUM-PUMC000373, Beijing, China) was cultured in Dulbecco’s modified Eagle medium (10569010, Gibco, Carlsbad, CA) appended to 10% fetal bovine serum (cat#10100147, Gibco) and penicillin mixture (working concentration of penicillin was 100 U/mL, streptomycin sulfate was 0.1 mg/mL, cat#P1400, Solarbio, Beijing, China) in an incubator with 5% CO_2_ at 37 °C. After culture for 1–2 passages, IL-32 (200 ng/mL; R&D Systems, Minneapolis, Minn.) was utilized for 48-h of cell treatment. The cells were then collected for the follow-up experiments.

Logarithmically growing cells were detached with 1 mL 0.25% trypsin (25200056, Gibco) for 3 min, and then the detachment was terminated utilizing the medium containing serum. Following cell concentration adjustment (1 × 10^5^ cells/mL), the cells were seeded into a 6-well plate with a glass slide for 24-h of conventional culture. Under above 75% confluence, cell transfection was implemented by referring to Lipofectamine 2000 (Invitrogen, Carlsbad, CA) utilizing shRNA (sh)-negative control (NC), sh-JAK1 (JAK1 knockdown), vector (NC for JAK1 overexpression), or overexpression (oe)-JAK1. The plasmids were constructed by GenePharma (Shanghai, China) and the plasmid concentration was 50 ng/mL. The cells were collected after 48-h transfection for subsequent experiments.

### Reverse transcription-quantitative polymerase chain reaction (RT-qPCR)

Total RNA extraction was processed by RNeasy Mini Kit (Qiagen, Valencia, CA). For mRNA determination, the complementary DNA (cDNA) was obtained by means of a RT Kit (RR047A, Takara, Otsu, Shiga, Japan). For miRNA determination, the miRNA First Strand cDNA SyntH&Esis (Tailing Reaction) kit (B532451-0020, Sangon, Shanghai, China) was adopted. SYBR Premix EX Taq kit (RR420A, Takara) was used to mix and load samples. The samples were subjected to RT-qPCR in a real-time fluorescence qPCR instrument (Bio-Rad CFX96, Bio-Rad Laboratories, Hercules, CA). The primers were synthesized by Sangon and displayed in Additional file 5: Tables S1 and S2. The relative expression of the product was calculated by 2^−ΔΔCt^ method with glyceraldehyde-3-phosphate dehydrogenase (GAPDH) as a normalizer for mRNA and U6 for miRNA.

### Western blot analysis

Tissue and cell samples were lysed with enhanced radioimmunoprecipitation assay lysis containing protease inhibitor (1 mM, cat# 36978, Thermo Fisher Scientific, Rockford, IL), and then the protein concentration was determined by BCA protein quantitative Kit (Boster, Wuhan, China). Following electrophoresis separation, the protein was transferred to a polyvinylidene fluoride membrane which was sealed with 5% bovine serum albumin at ambient temperature for 2 h to block the nonspecific binding. Then, overnight incubation of membrane with diluted primary rabbit antibodies was performed: JAK1 (ab133666, 1: 1000, Abcam, Cambridge, UK), phosphorylated (p)-JAK1 (#74129, 1: 1000, Cell Signaling Technology [CST], Danvers, MA), p-STAT1 (#9167, 1: 1000, CST), STAT1 (ab230428, 1: 1000, Abcam), STAT3 (ab68153, 1: 1000, Abcam), p-STAT3 (ab76315, 1: 1000, Abcam), Dicer 1 (ab14601, 1: 2000, Abcam), DGCR8 (ab191875, 1: 1000, Abcam), Drosha (ab12286, 1: 10,000, Abcam), and GAPDH (ab8245, 1: 5000, Abcam, normalizer). The following day, the membrane was reacted with horseradish peroxidase-labeled goat anti-rabbit secondary antibody (ab205719; 1: 2000; Abcam) or rabbit anti-mouse secondary antibody (ab6728, 1: 1000, Abcam) at ambient temperature for 1 h and then detected with enhanced chemiluminescence solution (EMD Millipore, Billerica, MA). Image J analysis software was utilized for quantifying the gray level of each band.

### Construction of mouse models

Seven-week-old male BALB/c mice (Hubei Provincial Center for Disease Control and Prevention,Wuhan, China) were fed adaptively at the temperature between 22 and 24 °C, under 12-h day/night cycles.

IL-32 transgenic (Tg) mice were generated [14] with the primer of: sense, 5ʹ-TGAGGAGCAGCACCCAGAGC-3ʹ, and antisense, 5ʹ-CCGTAGGACTGGAAAGAGGA-3ʹ. Genomic DNA samples were obtained from the tails of transgenic mice, followed by IL-32 gene expression determination utilizing RT-qPCR. IL-32 was not expressed in wild type (WT) mice. No overt phenotype was observed in IL-32-Tg mice compared with WT mice. The IL-32-Tg mice were viable and fertile, without tissue or organ abnormalities.

The construction process of phthalic anhydride (PA) model in mice was as follows: mice in the control group (n = 10) were treated with ddH_2_O, and mice in the PA group (n = 10) was treated with 100 μL (20 μL/cm^2^) of 5% PA. The mice were fed under this condition for 4 weeks, and then the next experiment was carried out.

MC903 model of AD in nude mice was constructed by using MC903 (Cayman, MI) according to the published experimental methods [15–17]. In brief, 2 nmol MC903 (20 μL dissolved in ethanol) was used on the back of ears of mice in the AD group (n = 10) for 14 days, and 20 μL ethanol was used for 14 days on mice in the control group (n = 10).

The ear thickness was evaluated utilizing a thickness gauge (Digimatic Indicator, Matusutoyo Co., Tokyo, Japan) to test the degree of skin inflammation caused by PA or MC903 treatment. To assess the severity of the PA-induced or MC903-induced AD, the clinical scores of mice in each group were scored as 0–6 points based on the average score of erythema (redness), scaly, itching and other symptoms, suggestive of the successful establishment of PA mouse model. At the end of the study, skin tissues and blood samples were collected. The skin-draining lymph nodes were obtained from the euthanized mice and weighed.

### In vivo experiment

PA-IL32-AD modeled mice were randomized into three groups (control, sh-JAK1, miR-155 inhibitor), with 10 mice in each group. The control and inhibitors were special oligonucleotide sequences, which were synthesized by Beomic Biotechnology Co., Ltd. (Jiangsu, China). The constructed liposome-coated lentiviral vector was injected into mice via tail vein at 25 μg/mouse. After that, the mice in each group were routinely cultured for 7 days.

### AD-reconstructed human epidermis (RHE) and corresponding treatment

RHE (0.33 cm^2^; 17 day) was purchased from Episkin (Lyon Cedex, France). This standard model was formed by the growth of human keratinocytes on an inert polycarbonate filter with chemical properties at the gas–liquid interface, with histological characteristics similar to that of real human epidermis. Briefly, an inflammatory AD cocktail constitutes 30 ng/mL of IL-4, 30 ng/mL of IL-13, and 3.5 ng/mL of tumor necrosis factor-α (TNF-α; PeproTech Inc, NJ) in the presence or absence of recombinant human IL-32 (100 ng/mL; YbdY Biotech, Seoul, Korea) was supplemented to the medium for 6-day culture. The culture medium was renewed every 48 h. After overnight incubation, the medium was renewed and the following treatments were started (all of them were treated with 6 independent reconstituted skin in vitro for 24 h):

- control: The AD-RHE model was treated with PBS;
- sh-JAK1: The AD-RHE model was treated with sh-JAK1;
- miR-155-inhibitor: The AD-RHE model was treated with miR-155 inhibitor;
- IL-32: The AD-RHE model was treated with IL-32 and the IL-32 gene fragment was transferred into the AD-RHE model with the same procedure as the construction of IL-32-Tg mice;

The sequence of sh-JAK1 was 5ʹ-CCAUCACUGUUGAUGACAAdTdT-3′, and the sequence of miR-155 inhibitor was 5′-ACCCCUAUCACGAUUAGCAUUAA-3′. The control and inhibitor were special oligonucleotide sequences (Biomics, Nantong, China). The constructed liposome-coated lentiviral vector was injected into mice through the tail vein at 25 μg/mouse (n = 10). In the end, the collected culture was frozen and stored at − 20 °C. RHE tissues were used for histological and immunohistochemical analyses.

### Enzyme-linked immunosorbent assay (ELISA)

In this study, sandwich ELISA was used to detect IL-32 expression [8]. The absorbance value was determined at 450 nm, and the concentration was calculated according to the standard.

### Hematoxylin and eosin (HE) staining

For pathological observation, the ear skin of mice was embedded in paraffin for 24 h, and then fixed by a series of dehydration and rehydration. The paraffin-embedded tissues were cut into 4 mm sections, and then stained with HE. The sections were observed by microscopy (LAS; Leica Microsystems, Buffalo grove, IL) and five visual fields were randomly selected for evaluation.

### Immunohistochemical staining

EnVision (EnVision + , Dako, Carpentaria, CA) system was used for immunohistochemical detection of human and mouse skin tissues. In brief, the skin tissue samples were shaved and rehydrated in double distilled water. Next, 3% H_2_O_2_ was used to block endogenous peroxidase activity, and then sodium citrate solution was used for antigen repair. Then, antibodies to IL-32 (ab37158, 1: 100, Abcam), JAK1 (ab125051, 1: 100, Abcam), p-JAK1 (PA5-104554, 1: 100, Invitrogen), IL-32 (ab37158, 1: 100, Abcam), p-STAT1 (#9167, 1: 800, Cell Signaling Technology), STAT1 (ab230428, 1: 100, Abcam), STAT3 (ab68153, 1: 100, Abcam), and p-STAT3 (ab76315, 1: 100, Abcam) were used to incubate the samples at 37 °C for 3 h. Then, the sample was further reacted with secondary antibody obtained from the Zhongshan Biotechnology company (Beijing, China). After diaminobenzidine staining, routine staining was performed followed by microscopic examination.

### Masson's trichrome staining

Skin tissue slices of mice in each group were dewaxed, stained with Weigert iron hematoxylin for 5–10 min, differentiated in acidic ethanol for 5–15 s, and treated with Masson for 3–5 min to return blue in color. Ponceau S Fuchsin staining solution was applied to slices for 5–10 min. Weak acidic working solution was prepared by mixing distilled water and weak acidic solution at a ratio of 2:1 and then used to wash slices for 1 min, which were rinsed with phosphomolybdic acid solution for another 1–2 min. Aniline blue staining solution was applied to slices for 1–2 min. Slices were then dehydrated with 95% ethanol and absolute ethanol, cleared with xylene, and sealed with neutral balsam. Nucleus and collagen fiber/protein were stained in blue while cytoplasm, muscle, and red blood cells were in red.

### Statistical analysis

Data analysis was processed utilizing the SPSS 21.0 statistical software (IBM, Armonk, NY). Each experiment was repeated three times independently. The measurement data were summarized by mean ± standard deviation. Data between two groups were compared employing independent sample *t* test, and those among multiples utilizing one-way analysis of variance (ANOVA), combined with Tukey’s post hoc tests. *p* < 0.05 indicated statistically significant difference.

## Results

### IL-32 is upregulated in AD

**Fig. 1:**
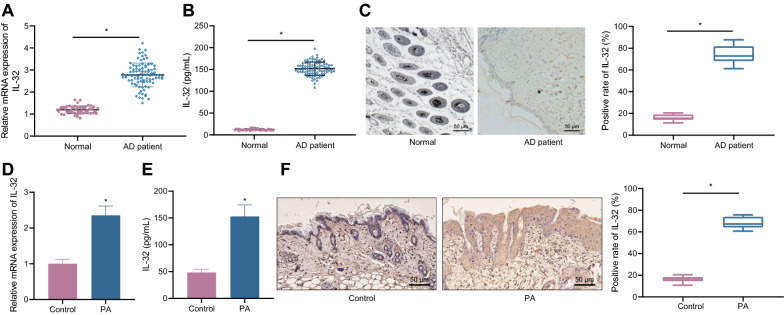
IL-32 is upregulated in AD. **A** The expression of IL-32 in clinical sample skin tissues by RT-qPCR (normal = 50, AD = 90). **B** Detection of IL-32 content in clinical blood samples by ELISA (normal = 50, AD = 90). **C** The expression level of IL-32 in clinical tissue samples by immunohistochemical staining (normal = 10, AD = 10, 50 μm). **D** Detection of IL-32 mRNA expression in mouse skin tissues by RT-qPCR (n = 10). **E** Detection of IL-32 content in mouse blood by ELISA (n = 10). **F** Immunohistochemical staining was used to detect the expression of IL-32 in mouse skin (50 μm, n = 10). * *p* < 0.05 vs. normal/control

As described by RT-qPCR, upregulation of IL-32 occurred in clinical skin tissues of patients with AD compared with normal individuals (Fig. 1A). Similar upregulation of IL-32 was detected in clinical blood samples by ELISA (Fig. 1B). Immunohistochemical staining showed that the expression of IL-32 in skin tissues of patients with AD was elevated than that in normal skin tissues (Fig. 1C). To study the effect of IL-32 on AD, PA specific dermatitis mouse model (model index evaluation is shown in Additional file 1: Fig. S1) was prepared followed by expression determination. It was indicated that the expression of IL-32 in skin tissues of PA-treated mice increased (Fig. 1D). ELISA results also identified elevated IL-32 expression in blood of PA-induced mice (Fig. 1E). Immunohistochemical staining clarified an increase in IL-32 expression in skin tissues of PA-treated mice (Fig. 1F).

The results show that IL-32 is elevated in skin tissues and blood sample of AD.

### IL-32 induces the occurrence of AD

**Fig. 2:**
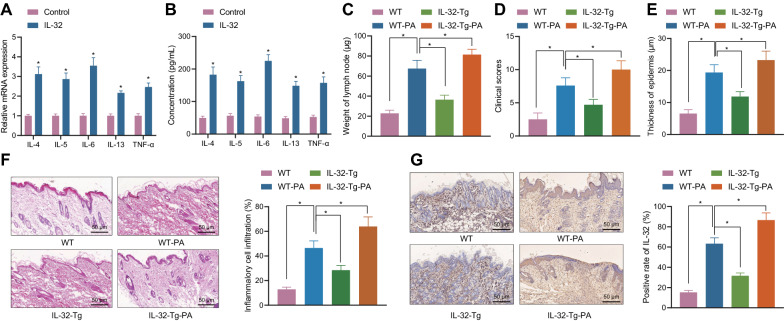
IL-32 promotes the occurrence of AD. **A** RT-qPCR was used to detect the expression of IL-4, IL-5, IL-6, IL-13 and TNF-α in HaCaT cell line (* *p* < 0.05 vs*.* control). B, ELISA detection of IL-4, IL-5, IL-6, IL-13 and TNF-α in HaCaT cell line (* *p* < 0.05 vs. control). **C** Weight of lymph nodes before and after PA treatment (n = 10, * *p* < 0.05). **D** Clinical scores of dorsal skin. **E** Thickness of epidermis. **F** HE staining for IL-32 in epidermal cells of mouse model before and after PA treatment and quantitative results (50 μm, n = 10, * *p* < 0.05). **G** Immunohistochemical staining for IL-32 of epidermal cells in mouse model before and after PA treatment and quantitative results (50 μm, n = 10, * *p* < 0.05). Cell experiment was repeated for three times

For further studying the role of IL-32 in AD, we used RT-qPCR to detect the levels of IL-4, IL-5, IL-6, IL-13, and TNF-α in HaCaT cells before and after treatment with IL-32. It was noted that the mRNA expression of IL-4, IL-5, IL-6, IL-13 and TNF-α in IL-32-induced HaCaT cells was increased (Fig. 2A). ELISA results showed the similar results as RT-qPCR (Fig. 2B). IL-32-Tg mice (model index evaluation is shown in Additional file 2: Fig. S2A) were further prepared and induced with PA. Increased weight of lymph nodes were observed in the IL-32-Tg-control mice relative to WT control mice, while IL-32-Tg mice exhibited higher weight of lymph nodes compared with WT control mice in the presence of PA (Fig. 2C).

Analysis of clinical scores (Fig. 2D) and thickness of epidermis (Fig. 2E) showed that the dermatitis was severer and epidermis was thicker in IL-32-Tg-control mice than that in WT control mice; similar changing tendency was observed in comparison between the IL-32-Tg-PA mice and WT-PA mice as well as between PA-induced mice and mice without PA treatment. Additionally, the results of HE staining (Fig. 2F) and immunohistochemical staining (Fig. 2G) showed that the cell infiltration was more obvious and expression of IL-32 was elevated in IL-32-Tg-PA mice; the degree of epidermal cell infiltration and IL-32 expression in WT-PA mice were weaker than those in IL-32-Tg-PA mice, but stronger than those in IL-32-Tg-control mice; the degree of inflammatory infiltration and the expression of IL-32 in WT control mice were the weakest among those treated mice.

These results suggest that IL-32 aggravates AD through the specific epidermis-related factors.

### IL-32 promotes JAK1 expression and activates its downstream signaling pathway in AD

**Fig. 3:**
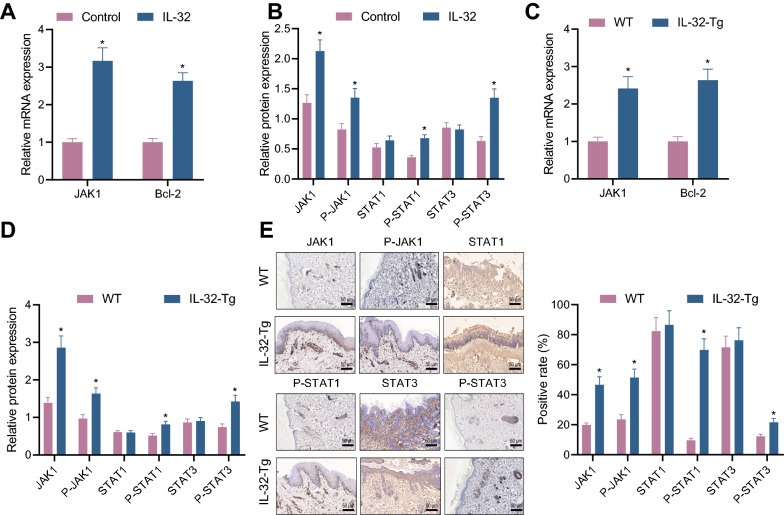
IL-32 promotes JAK1 expression and activates its downstream signaling pathway in AD. **A** The expression levels of JAK1 and Bcl-2 in HaCaT cell lines were detected by RT-qPCR. **B** The protein levels of JAK1, p-JAK1, p-STAT1/3 and STAT1/3 in HaCaT cell lines were detected by Western blot analysis. **C** The expression levels of JAK1 and Bcl-2 in mouse skin tissues were detected by RT-qPCR (n = 10). **D** The protein levels of JAK1, p-JAK1, p-STAT1/3 and STAT1/3 in mouse skin tissues were detected by Western blot analysis (n = 10). **E** Immunohistochemical detection of JAK1, p-JAK1, p-STAT1/3 and STAT1/3 in mouse skin tissues (50 μm). * *p* < 0.05 vs. control/WT. Cell experiment was repeated for three times

As earlier reported, IL-32 exerts its intracellular regulatory function by activating the JAK signaling pathway [18, 19]. We then focused on whether overexpression of IL-32 in AD affects the JAK signaling pathway. The results demonstrated that treatment with IL-32 brought about elevations in expression of JAK1, Bcl-2, p-JAK1, and p-STAT1/3 in HaCaT cell lines, but exerted no function in STAT1/3 protein level (Fig. 3A, B, Additional file 3: Fig. S3A). Furthermore, we noted an enhancement in the mRNA expression of JAK1 and Bcl-2 in skin tissues of IL-32-Tg mice relative to WT mice (Fig. 3C). Western blot and immunohistochemistry results showed that the protein levels of JAK1, p-JAK1 and p-STAT1/3 in IL-32-Tg mice were notably higher than those in WT mice, while STAT1/3 protein level did not differ significantly (Fig. 3D, E, Additional file 3: Fig. S3B).

These results suggest that IL-32 upregulates JAK1 expression in AD, and then promotes the phosphorylation level of downstream genes of the JAK1 signaling pathway to activate intracellular JAK signaling pathway.

### Overexpression of JAK1 promotes the expression of miR-155 in AD

**Fig. 4:**
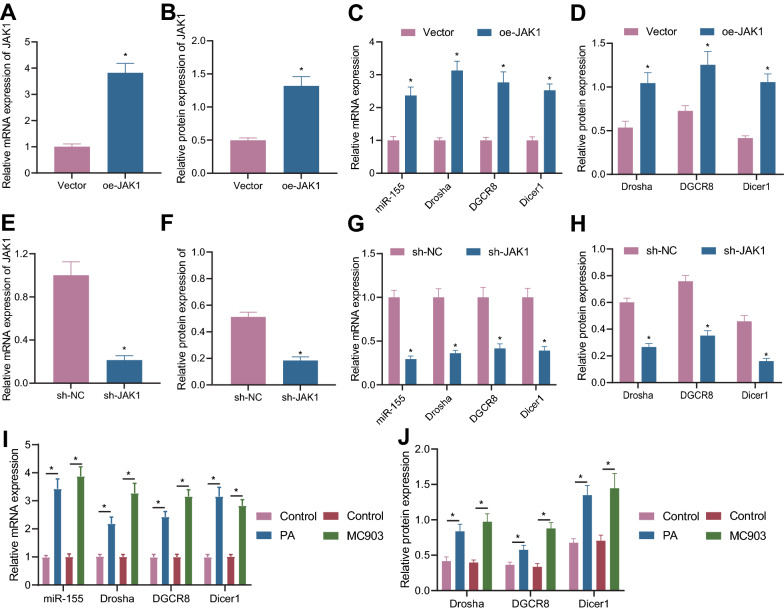
Overexpression of JAK1 promotes the expression of miR-155 in AD. **A** The expression level of JAK1 in HaCaT cell line following oe-JAK1 treatment was detected by RT-qPCR (* *p* < 0.05 vs. vector). **B** JAK1 protein level in HaCaT cell line following oe-JAK1 treatment was detected by Western blot analysis. **C** The expression levels of miR-155, Drosha, DGCR8 and Dicer1 in HaCaT cell line following oe-JAK1 treatment were detected by RT-qPCR (* *p* < 0.05 vs. vector). **D** The protein levels of Drosha, DGCR8 and Dicer1 in HaCaT cell line following oe-JAK1 treatment were detected by Western blot analysis. **E** The expression of JAK1 in HaCaT cell line following sh-JAK1 treatment was detected by RT-qPCR (* *p* < 0.05 vs. sh-NC). **F** JAK1 protein level in HaCaT cell line following sh-JAK1 treatment was detected by Western blot analysis. **G** The expression level of miR-155, Drosha, DGCR8 and Dicer1 in HaCaT cell line following sh-JAK1 treatment was detected by RT-qPCR (* *p* < 0.05 vs. sh-NC). **H** The protein expression levels of Drosha, DGCR8 and Dicer1 in HaCaT cell line following sh-JAK1 treatment were detected by Western blot analysis. **I** The expression levels of miR-155, Drosha, DGCR8 and Dicer1 in skin tissues of mice with AD induced by PA or MC903 were detected by RT-qPCR (n = 10, * *p* < 0.05 vs. control). **J** Western blot analysis was used to detect the protein levels of Drosha, DGCR8 and Dicer1 in skin tissues of mice with AD induced by PA or MC903. Cell experiment was repeated for three times

The interaction between JAK1/2 kinase and miR-155 has been documented [20]. In the present study, we attempted to study the regulatory role of JAK1 in miR-155. As shown in RT-qPCR and Western blot analyzes, oe-JAK1-transfected HaCaT cell lines had increased levels of JAK1, miR-155, Drosha, DGCR8, and Dicer1 (Fig. 4A–D, Additional file 3: Fig. S3C, D), while sh-JAK1 treatment led to opposite results (Fig. 4E–H, Additional file 3: Fig. S3E–G).

Moreover, in vivo assay also demonstrated that expression of miR-155, Drosha, DGCR8 and Dicer1 in skin tissues of AD mice induced by PA and MC903 was increased (Fig. 4I, J). Further, levels of miR-155, Drosha, DGCR8 and Dicer1 were upregulated in skin tissues of IL-32-Tg mice compared with WT mice (Additional file 2: Fig. S2B, C).

Thus, overexpression of JAK1 elevates the expression of miR-155, Drosha, DGCR8 and Dicer1 in AD.

### Il-32/JAK1/miR-155 axis regulates inflammation in AD-RHE model

**Fig. 5:**
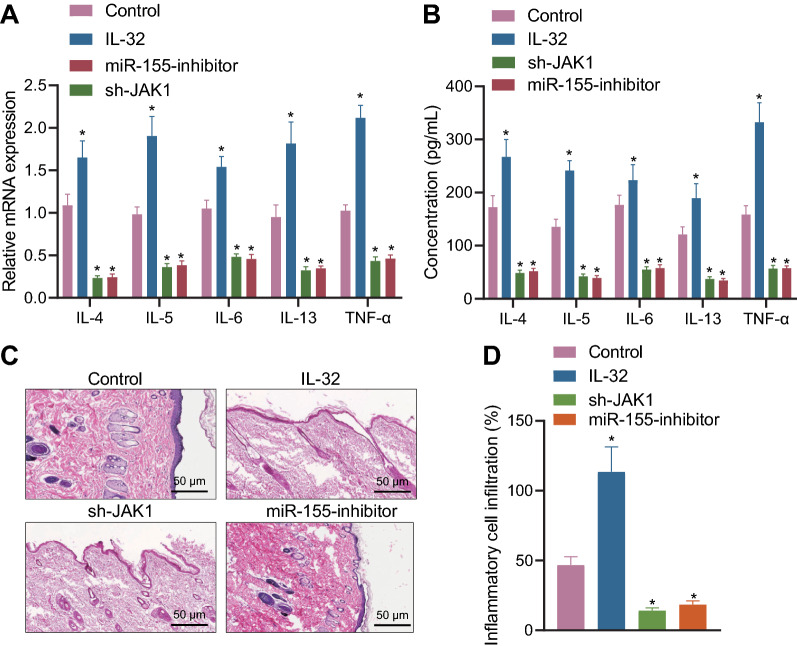
IL-32/JAK1/miR-155 axis regulates inflammation in AD-RHE model. **A** RT-qPCR was used to detect the expression levels of IL-4, IL-5, IL-6, IL-13 and TNF-α in AD-RHE model. **B** ELISA was used to detect the expression levels of IL-4, IL-5, IL-6, IL-13 and TNF-α in AD-RHE model. **C** Representative images for skin inflammation clinical scores (left) and HE staining (right) for AD-RHE model (50 μm). **D** Quantitative analysis of HE results. n = 10. * *p* < 0.05 vs control

AD-RHE model was then prepared for the following experimentations. As expected, IL-32 treatment induced increases in levels of IL-32, JAK1 and miR-155, while silencing of JAK1 reduced the expression of JAK1 and miR-155 with no marked difference in IL-32 expression; meanwhile, inhibition of miR-155 diminished expression of miR-155, with no alteration in the expression of IL-32 and JAK1, indicating successful transfection (Additional file 4: Fig. S4). RT-qPCR results highlighted that IL-32 treatment induced increases in the mRNA expression of IL-4, IL-5, IL-6, IL-13 and TNF-α, which opposing tendency was seen in response to sh-JAK1 or miR-155 inhibitor, but no significant difference was witnessed in comparison between sh-JAK1 and miR-155 inhibitor treatment (Fig. 5A). ELISA results turned out to be similar to RT-qPCR results (Fig. 5B). HE staining revealed that sh-JAK1 or miR-155 inhibitor treatment significantly reduced the inflammatory degree, while opposite trends were observed following IL-32 treatment in AD-RHE model (Fig. 5C, D).

### IL-32 promotes the development of AD through the JAK1/miR-155 axis

**Fig. 6:**
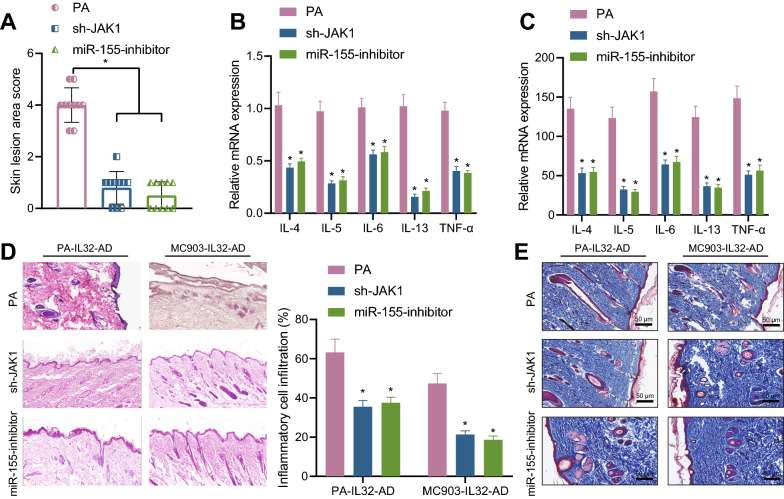
IL-32 promotes the development of AD through the JAK1/miR-155 axis. **A** The mouse skin inflammation clinical scores. **B** The expression levels of IL-4, IL-5, IL-6, IL-13 and TNF-α in skin tissues in the PA-IL-32-AD-model were detected by RT-qPCR. **C** The content of IL-4, IL-5, IL-6, IL-13 and TNF-α skin tissues in the PA-IL-32-AD-model was detected by ELISA. **D** HE staining of PA-IL-32-AD-model and MC903-IL-32-AD-model (50 μm). **E** Skin tissue healing of PA-IL-32-AD-model and MC903-IL-32-AD-model detected by Masson's trichrome staining (50 μm). n = 10. * *p* < 0.05 vs. control

PA-IL-32-AD-model was further subjected to different treatment in vivo, results of which showed that sh-JAK1 or miR-155 inhibitor effectively alleviated the development of specific dermatitis in PA-IL-32-AD-model, based on the skin inflammation clinical scores (Fig. 6A). RT-qPCR and ELISA showed that the levels of IL-4, IL-5, IL-6, IL-13 and TNF-α in PA-IL-32-AD-model treated with sh-JAK1 or miR-155 inhibitor were significantly decreased (Fig. 6B, C). HE staining results showed that sh-JAK1 or miR-155 inhibitor significantly reduced the inflammatory degree in PA-IL-32-AD-model and MC903-IL-32-AD-model (Fig. 6D). Masson's trichrome staining was performed to evaluate the dermal collagen accumulation to assess skin tissue healing (Fig. 6E). It was found that collagen fibers in the papillary layer of the skin tissues were sparse, and a large number of inflammatory cells were observed in the control group. In addition, there were dense collagen fibers and fiber bundles as well as fewer inflammatory cells in the skin tissues of mice in the PA-IL32-AD-model group and MC903-IL32-AD-model group treated with sh-JAK1 and miR-155 inhibitor.

These results suggest that IL-32 promotes the development of AD by activating the JAK1/miR-155 axis.

## Discussion

It is known that AD has become a health burden throughout the world [21]. Our current study emphasized that IL-32 facilitated the occurrence of AD by regulating the JAK1/miR-155 axis.

In the first place, we successfully established PA and MC903 mouse models. PA is a well-studied compound that induces allergic dermatitis in mice to establish an AD model [22]. MC903, a low-calcemic analog of vitamin D3, alters skin morphology and inflammation, and increase in serum IgE levels was observed in MC903-treated mice, highly suggestive of its potential for investigating immunologic abnormalities in AD development [23]. Moreover, we found in this study that IL-32 was elevated in AD and promoted the occurrence of AD. Mounting evidence has documented the promoting role of IL-32 in the progression of AD. For example, suppressed expression of IL-32 by rutin alleviated the development of AD [24]. Additionally, IL-32 induced by dermatophagoides farinae extract and 2,4-dinitrochlorobenzene aided in aggravating AD-like symptoms [25]. Likewise, promoted induction of IL-32 is found in hair follicle-derived keratinocytes collected from donors with AD [26].

Furthermore, we demonstrated that IL-32 promoted JAK1 expression and thus activated its downstream signaling pathway in AD. Of note, previous studies have identified the interaction between IL-32 and JAK. For instance, IL-32 leads to activated JAK1, which promotes immune-mediated inflammation of rheumatoid arthritis [9]. Moreover, the interaction between IL-32 and the JAK/STAT signaling has been revealed in liver inflammation and fibrosis related to hepatitis C virus [18]. The therapeutic function of JAK1 has been increasingly highlighted. Notably, Baricitinib, an oral JAK inhibitor that is capable of repressing JAK1, can be used to treat many skin diseases including AD [27] and can ameliorate the symptoms of moderate-to-severe AD [28]. In addition, JAK1, with enrichment of AD-associated rare coding variants, was suggested to be promising for systemic AD therapy [29].

Another crucial finding was that JAK1 increased the expression of miR-155 in AD to promote its development. Intriguingly, the regulatory relationship between JAK and miR-155 has been revealed by several studies. As previously reported, the activation of JAK/STAT signaling pathway by inflammatory cytokines increases the expression of miR-155 in human retinal pigment epithelial cells [30]. In addition, inhibition of JAK results in downregulation of miR-155 expression in cutaneous T-cell lymphoma [30]. Furthermore, inhibition of the JAK/STAT signaling by methylprednisolone diminishes miR-155 expression in T cells, thereby exerting an anti-inflammatory role [31]. In consistency with our result, many studies have documented the participation of miR-155 in the progression of AD. As previously reported, miR-155 is significantly highly expressed in patients with AD and might result in chronic skin inflammation by elevating the proliferative response of T(H) cells via repression of CTLA-4 [32]. Additionally, miR-155 is accountable for the pathogenesis of AD by regulating the differentiation of T helper type 17 (Th17) cells [33]. Moreover, silencing of miR-155-5p alleviates the thickening of the epidermis in AD while diminishing the inflammatory cell infiltration and Th2 cytokine secretion [34]. Therefore, it is demonstrated that the role of IL-32 in AD was achieved through regulation of the JAK1/miR-155 axis.

In the present study, we identified that IL-32 promoted the development of AD, increased the expression of JAK1 and thus activated its downstream factor miR-155 to facilitate the occurrence of AD. However, there are still some open questions needed to be solved, including the downstream targets of miR-155 in IL-32-mediated AD occurrence, the detailed mechanism underlying JAK1 activating miR-155, and whether JAK1 inhibitors have therapeutic effect on AD development. Further study would be focused on these significant questions in the future.

## Conclusions

**Fig. 7:**
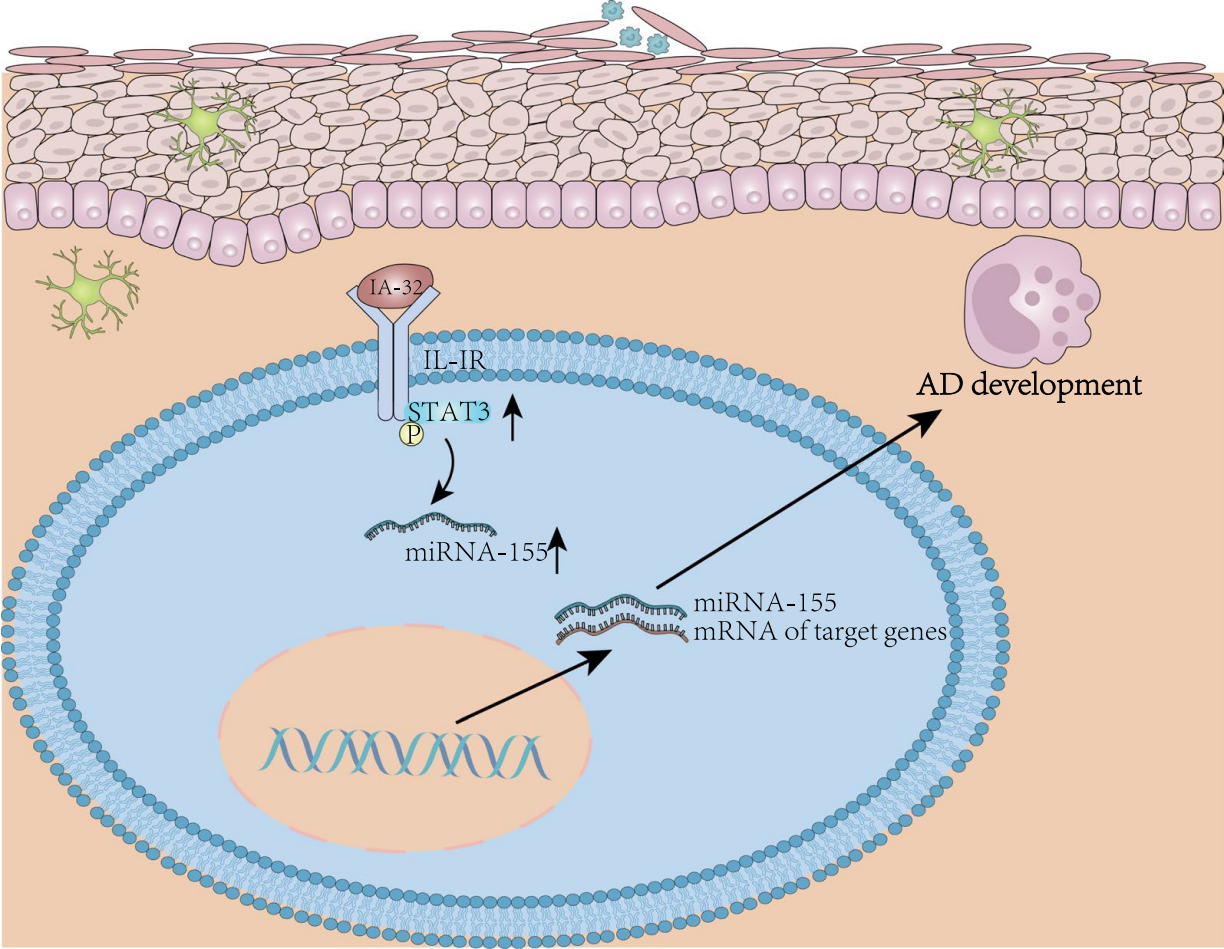
The molecular mechanism plot for the role of IL-32 in AD. Highly expressed IL-32 in AD upregulates JAK1 and activates its downstream signaling pathway, which increases the expression of miR-155, thus promoting the development of AD

In conclusion, the results obtained in the study reveal upregulation of IL-32 in AD increases JAK1 expression and thus activates its downstream signaling pathway, thereby promoting the expression of miR-155, which facilitates the development of AD (Fig. 7). This finding may offer a novel way for control of AD.

## Supplementary Information

**Additional file 1: Figure S1.** The model index evaluation in PA mouse model. A, Representative clinical features and corresponding HE staining of dorsal skin (50 μm). B, Statistics of ear thickness of PA model and WT mice. C, Clinical score, the whole body was divided into four parts: head and neck, upper limbs, trunk, and lower limbs. The percentages of the above parts in body surface area were 10%, 20%, 30% and 40%, respectively. The four sites were scored with the following criteria: 0 = no rash, 1 = 1–9%, 2 = 10–29%, 3 = 30–49%, 4 = 50–69%, 5 = 70–89%, 6 = 90–100%. To help assess the area involved, the neck was regarded as part of the head, the armpit and groin were regarded as part of the trunk, and the buttocks were regarded as part of the lower extremities. n = 10.

**Additional file 2: Figure S2.** The IL-32, miR-155, Drosha, DGCR8, and Dicer1 expression in IL-32-Tg mice or WT mice. A, IL-32 level in skin tissues of WT and IL-32-Tg mice detected using RT-qPCR. B, The expression levels of miR-155, Drosha, DGCR8, and Dicer1 in WT and IL-32-Tg mice analyzed using RT-qPCR. C: Western blot analysis of Drosha, DGCR8, and Dicer1 proteins levels in WT and IL-32-Tg mice; n = 10, * *p* < 0.05 vs. WT control mice.

**Additional file 3: Figure S3.** A, Representative protein bands of Fig. 3B. B, Representative protein bands of Fig. 3D. C, Representative protein bands of Fig. 4B. D, Representative protein bands of Fig. 4D. E, Representative protein bands of Fig. 4F. F, Representative protein bands of Fig. 4H. G, Representative protein bands of Fig. 4J.

**Additional file 4: Figure S4.** The expression of IL-32, JAK1 and miR-155 in AD-RHE mouse models treated with sh-JAK1 or miR-155 inhibitor detected using RT-qPCR; n = 10, * *p* < 0.05 vs. control mice.

**Additional file 5: Table S1.** The expression of IL-32, JAK1 and miR-155 in AD-RHE mouse models treated with sh-JAK1 or miR-155 inhibitor detected using RT-qPCR; n = 10, * *p* < 0.05 vs. control mice.

## Data Availability

The datasets generated and/or analysed during the current study are available in the manuscript and additional materials.

## Abbreviations

AD: Atopic dermatitis
IL-32: Interleukin-32
JAK1: Janus-activated kinase-1
miRs: MicroRNAs
sh: ShRNA
NC: Negative control
oe: Overexpression
RT-qPCR: Reverse transcription-quantitative polymerase chain reaction
cDNA: Complementary DNA
p: Phosphorylated
DGCR8: DiGeorge syndrome chromosomal region 8
GAPDH: Glyceraldehyde-3-phosphate dehydrogenase
PA: Phthalic anhydride
Tg: Transgenic
PA: Phthalic anhydride
RHE: Reconstructed human epidermis
ELISA: Enzyme-linked immunosorbent assay
HE: Hematoxylin and eosin
ANOVA: Analysis of variance
Th17: T helper type 17

## References

[1] Lipsky ZW, Marques CNH, German GK (2020). Lipid depletion enables permeation of *Staphylococcus aureus* bacteria through human stratum corneum. Tissue Barriers.

[2] Yan F, Li F, Liu J, Ye S, Zhang Y, Jia J (2020). The formulae and biologically active ingredients of Chinese herbal medicines for the treatment of atopic dermatitis. Biomed Pharmacother..

[3] Baghoomian W, Na C, Simpson EL (2020). New and emerging biologics for atopic dermatitis. Am J Clin Dermatol.

[4] Nahm DH (2015). Personalized immunomodulatory therapy for atopic dermatitis: an allergist's view. Ann Dermatol.

[5] Rojahn TB, Vorstandlechner V, Krausgruber T, Bauer WM, Alkon N, Bangert C (2020). Single-cell transcriptomics combined with interstitial fluid proteomics defines cell type-specific immune regulation in atopic dermatitis. J Allergy Clin Immunol.

[6] Jeong HJ, Shin SY, Oh HA, Kim MH, Cho JS, Kim HM (2011). IL-32 up-regulation is associated with inflammatory cytokine production in allergic rhinitis. J Pathol.

[7] Kitayama N, Otsuka A, Nonomura Y, Nakashima C, Honda T, Kabashima K (2017). Decrease in serum IL-32 level in patients with atopic dermatitis after cyclosporine treatment. J Eur Acad Dermatol Venereol.

[8] Meyer N, Zimmermann M, Burgler S, Bassin C, Woehrl S, Moritz K (2010). IL-32 is expressed by human primary keratinocytes and modulates keratinocyte apoptosis in atopic dermatitis. J Allergy Clin Immunol.

[9] Malemud CJ (2011). Dysfunctional immune-mediated inflammation in rheumatoid arthritis dictates that development of anti-rheumatic disease drugs target multiple intracellular signaling pathways. Antiinflamm Antiallergy Agents Med Chem.

[10] Dhillon S (2020). Delgocitinib: first approval. Drugs.

[11] Yu X, Wang M, Li L, Zhang L, Chan MTV, Wu WKK (2020). MicroRNAs in atopic dermatitis: a systematic review. J Cell Mol Med.

[12] Bergallo M, Accorinti M, Galliano I, Coppo P, Montanari P, Quaglino P (2020). Expression of miRNA 155, FOXP3 and ROR gamma, in children with moderate and severe atopic dermatitis. G Ital Dermatol Venereol.

[13] Tang X, Fu J, Tan X, Shi Y, Ye J, Guan W (2020). The miR-155 regulates cytokines expression by SOSC1 signal pathways of fish in vitro and in vivo. Fish Shellfish Immunol.

[14] Lee YS, Han SB, Ham HJ, Park JH, Lee JS, Hwang DY (2020). IL-32gamma suppressed atopic dermatitis through inhibition of miR-205 expression via inactivation of nuclear factor-kappa B. J Allergy Clin Immunol.

[15] Fu X, Hong C (2019). Osthole attenuates mouse atopic dermatitis by inhibiting thymic stromal lymphopoietin production from keratinocytes. Exp Dermatol.

[16] Lee DY, Hwang CJ, Choi JY, Park MH, Song MJ, Oh KW (2017). Inhibitory effect of carnosol on phthalic anhydride-induced atopic dermatitis via inhibition of STAT3. Biomol Ther (Seoul).

[17] Liu XJ, Mu ZL, Zhao Y, Zhang JZ (2016). Topical tetracycline improves MC903-induced atopic dermatitis in mice through inhibition of inflammatory cytokines and thymic stromal lymphopoietin expression. Chin Med J (Engl).

[18] Moschen AR, Fritz T, Clouston AD, Rebhan I, Bauhofer O, Barrie HD (2011). Interleukin-32: a new proinflammatory cytokine involved in hepatitis C virus-related liver inflammation and fibrosis. Hepatology.

[19] Radom-Aizik S, Zaldivar F, Leu SY, Galassetti P, Cooper DM (2008). Effects of 30 min of aerobic exercise on gene expression in human neutrophils. J Appl Physiol (1985)..

[20] Yan H, Wang S, Li Z, Zhao W, Wang Z, Sun Z (2016). Upregulation of miRNA-155 expression by OxLDL in dendritic cells involves JAK1/2 kinase and transcription factors YY1 and MYB. Int J Mol Med.

[21] Boguniewicz M (2020). Biologics for atopic dermatitis. Immunol Allergy Clin N Am.

[22] Lee YJ, Oh MJ, Lee DH, Lee YS, Lee J, Kim DH (2020). Anti-inflammatory effect of bee venom in phthalic anhydride-induced atopic dermatitis animal model. Inflammopharmacology.

[23] Moosbrugger-Martinz V, Schmuth M, Dubrac S (2017). A mouse model for atopic dermatitis using topical application of vitamin D3 or of its analog MC903. Methods Mol Biol.

[24] Choi JK, Kim SH (2013). Rutin suppresses atopic dermatitis and allergic contact dermatitis. Exp Biol Med (Maywood).

[25] Choi JK, Kim SH (2014). Inhibitory effect of galangin on atopic dermatitis-like skin lesions. Food Chem Toxicol.

[26] Yoshikawa Y, Sasahara Y, Takeuchi K, Tsujimoto Y, Hashida-Okado T, Kitano Y (2013). Transcriptional analysis of hair follicle-derived keratinocytes from donors with atopic dermatitis reveals enhanced induction of IL32 gene by IFN-gamma. Int J Mol Sci.

[27] Koumaki D, Koumaki V, Lagoudaki E, Bertsias G (2020). Palmoplantar pustulosis-like eruption induced by baricitinib for treatment of rheumatoid arthritis. Eur J Case Rep Intern Med..

[28] Napolitano M, Fabbrocini G, Cinelli E, Stingeni L, Patruno C (2020). Profile of baricitinib and its potential in the treatment of moderate to severe atopic dermatitis: a short review on the emerging clinical evidence. J Asthma Allergy.

[29] Mucha S, Baurecht H, Novak N, Rodriguez E, Bej S, Mayr G (2020). Protein-coding variants contribute to the risk of atopic dermatitis and skin-specific gene expression. J Allergy Clin Immunol.

[30] Kutty RK, Nagineni CN, Samuel W, Vijayasarathy C, Hooks JJ, Redmond TM (2010). Inflammatory cytokines regulate microRNA-155 expression in human retinal pigment epithelial cells by activating JAK/STAT pathway. Biochem Biophys Res Commun.

[31] Kopp KL, Ralfkiaer U, Gjerdrum LM, Helvad R, Pedersen IH, Litman T (2013). STAT5-mediated expression of oncogenic miR-155 in cutaneous T-cell lymphoma. Cell Cycle.

[32] Sonkoly E, Janson P, Majuri ML, Savinko T, Fyhrquist N, Eidsmo L (2010). MiR-155 is overexpressed in patients with atopic dermatitis and modulates T-cell proliferative responses by targeting cytotoxic T lymphocyte-associated antigen 4. J Allergy Clin Immunol.

[33] Ma L, Xue HB, Wang F, Shu CM, Zhang JH (2015). MicroRNA-155 may be involved in the pathogenesis of atopic dermatitis by modulating the differentiation and function of T helper type 17 (Th17) cells. Clin Exp Immunol.

[34] Wang X, Chen Y, Yuan W, Yao L, Wang S, Jia Z (2019). MicroRNA-155-5p is a key regulator of allergic inflammation, modulating the epithelial barrier by targeting PKIalpha. Cell Death Dis.

