# Supplementary figures and images for: IL-32 promotes the occurrence of atopic dermatitis by activating the JAK1/microRNA-155 axis
Source: J Transl Med. 2022 May 11;20:207. doi: 10.1186/s12967-022-03375-x (PMC9097387; doi:10.1186/s12967-022-03375-x)

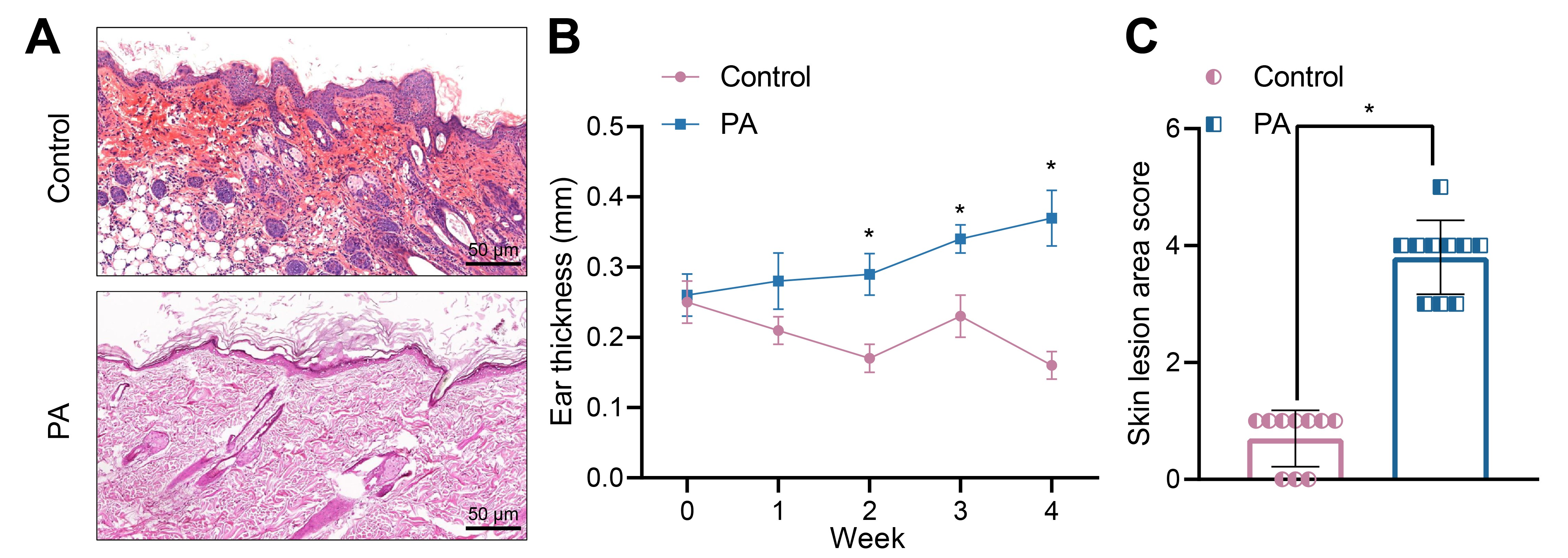

Supplement: Supplementary file 1 — Additional file 1: Figure S1. The model index evaluation in PA mouse model. A, Representative clinical features and corresponding HE staining of dorsal skin (50 μm). B, Statistics of ear thickness of PA model and WT mice. C, Clinical score, the whole body was divided into four parts: head and neck, upper limbs, trunk, and lower limbs. The percentages of the above parts in body surface area were 10%, 20%, 30% and 40%, respectively. The four sites were scored with the following criteria: 0 = no rash, 1 = 1–9%, 2 = 10–29%, 3 = 30–49%, 4 = 50–69%, 5 = 70–89%, 6 = 90–100%. To help assess the area involved, the neck was regarded as part of the head, the armpit and groin were regarded as part of the trunk, and the buttocks were regarded as part of the lower extremities. n = 10. [file 12967_2022_3375_MOESM1_ESM.jpg]

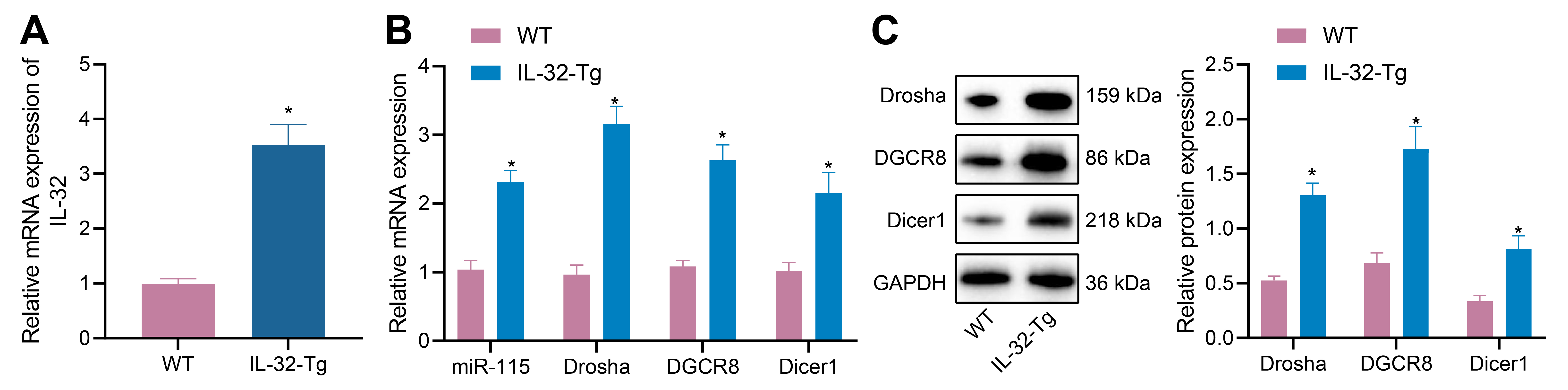

Supplement: Supplementary file 2 — Additional file 2: Figure S2. The IL-32, miR-155, Drosha, DGCR8, and Dicer1 expression in IL-32-Tg mice or WT mice. A, IL-32 level in skin tissues of WT and IL-32-Tg mice detected using RT-qPCR. B, The expression levels of miR-155, Drosha, DGCR8, and Dicer1 in WT and IL-32-Tg mice analyzed using RT-qPCR. C: Western blot analysis of Drosha, DGCR8, and Dicer1 proteins levels in WT and IL-32-Tg mice; n = 10, * p < 0.05 vs. WT control mice. [file 12967_2022_3375_MOESM2_ESM.jpg]

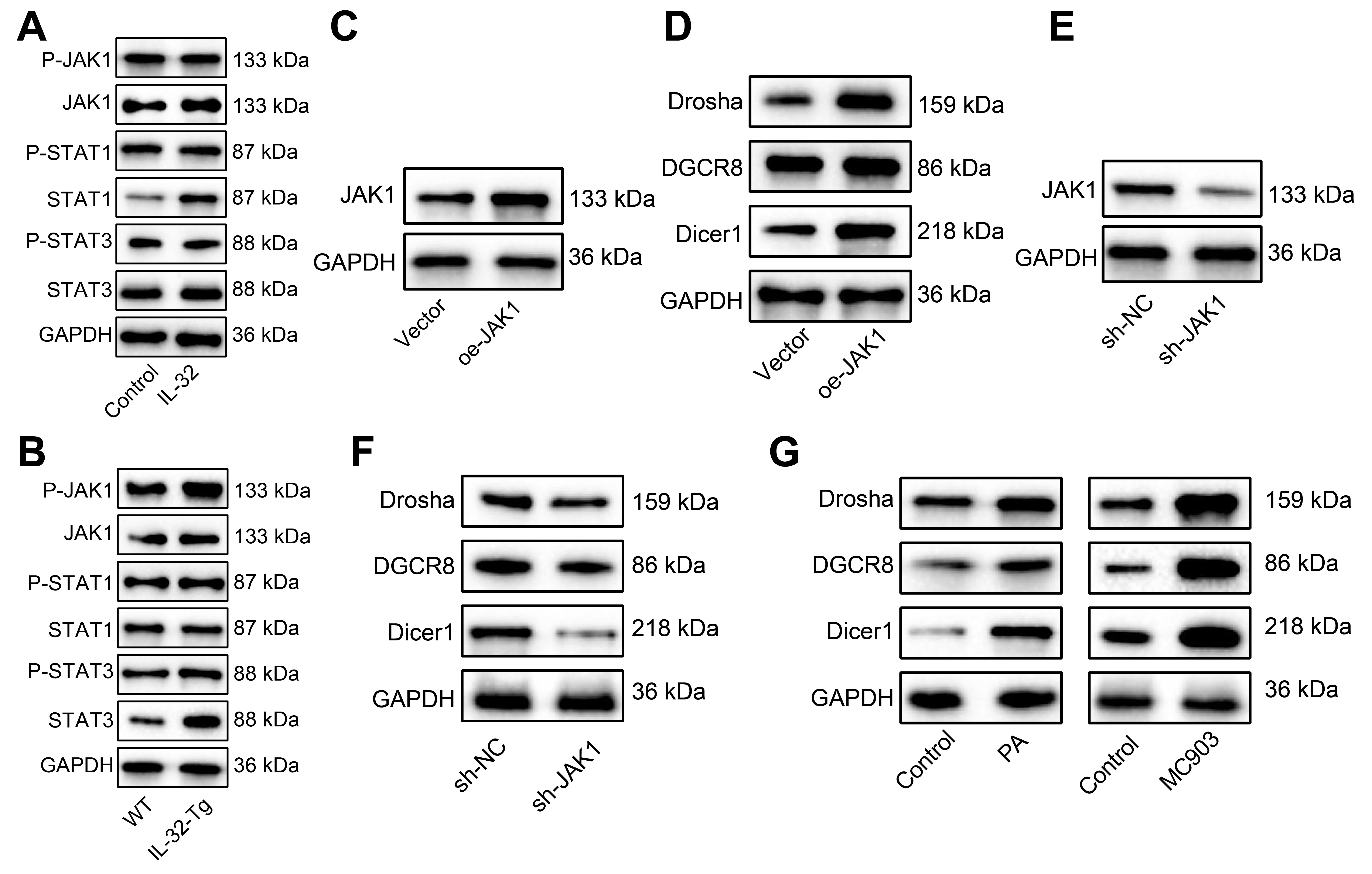

Supplement: Supplementary file 3 — Additional file 3: Figure S3. A, Representative protein bands of Fig. 3B. B, Representative protein bands of Fig. 3D. C, Representative protein bands of Fig. 4B. D, Representative protein bands of Fig. 4D. E, Representative protein bands of Fig. 4F. F, Representative protein bands of Fig. 4H. G, Representative protein bands of Fig. 4J. [file 12967_2022_3375_MOESM3_ESM.jpg]

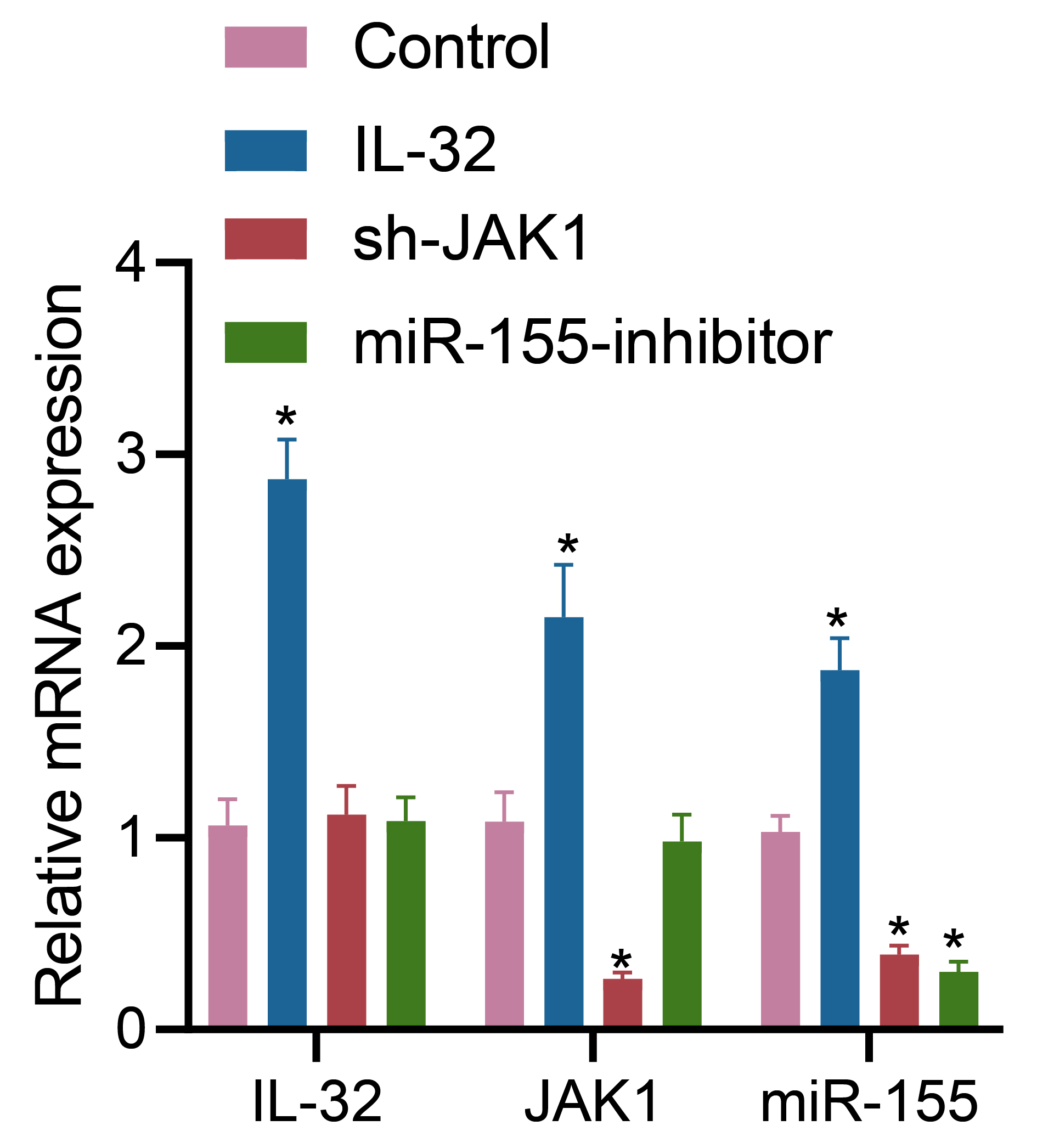

Supplement: Supplementary file 4 — Additional file 4: Figure S4. The expression of IL-32, JAK1 and miR-155 in AD-RHE mouse models treated with sh-JAK1 or miR-155 inhibitor detected using RT-qPCR; n = 10, * p < 0.05 vs. control mice. [file 12967_2022_3375_MOESM4_ESM.jpg]
