# Supplementary material for: IL-32 promotes the occurrence of atopic dermatitis by activating the JAK1/microRNA-155 axis
Source: J Transl Med. 2022 May 11;20:207. doi: 10.1186/s12967-022-03375-x (PMC9097387; doi:10.1186/s12967-022-03375-x)
Supplement: Supplementary file 5 — Additional file 5: Table S1. The expression of IL-32, JAK1 and miR-155 in AD-RHE mouse models treated with sh-JAK1 or miR-155 inhibitor detected using RT-qPCR; n = 10, * p < 0.05 vs. control mice. [file 12967_2022_3375_MOESM5_ESM.docx]

**Table S1** Primer sequences for RT-qPCR (human)

| Gene | Sequence | |
| --- | --- | --- |
| IL-32 | Forward | 5'-TGAGGAGCAGCACCCAGAGC-3' |
|  | Reverse | 5'-CCGTAGGACTGGAAAGAGGA-3' |
| IL-4 | Forward | 5'-CCGTAACAGACATCTTTGCTGCC-3' |
|  | Reverse | 5'-GAGTGTCCTTCTCATGGTGGCT-3' |
| IL-5 | Forward | 5′-GATAGCCAATGAGACTCTGAGG-3′ |
|  | Reverse | 5′-TTGCCTGGAGGAAAATACTT-3′ |
| IL-6 | Forward | 5′-ATGAACTCCTTCTCCACAAGCGC-3′ |
|  | Reverse | 5′-GAAGAGCCCTCAGGCTGGACTG-3′ |
| IL-13 | Forward | 5'-CCT CAA TCC TCT CCT GTT GG-3' |
|  | Reverse | 5'-TTT GGT GTC TCG GAC ATG C-3' |
| TNF-α | Forward | 5′-CTCTTCTGCCTGCTGCACTTTG-3′ |
|  | Reverse | 5′-ATGGGCTACAGGCTTGTCACTC-3′ |
| JAK1 | Forward | 5’- CTCACCAGGATGCGGATAAA-3’ |
|  | Reverse | 5’-AGTTTCCAAGGTAGCCAAGTAT-3’ |
| STAT1 | Forward | 5’-GACCCAATCCAGATGTCTATGA-3’ |
|  | Reverse | 5’-CCCGACTGAGCCTGATTA-3’ |
| miR-155 | Forward | 5′-CTGGGTTAATGCTAATCGTGAT-3′ |
|  | Reverse | Reverse Universal Primer |
| Drosha | Forward | 5’-CTGTCGATGCACCAGATT-3’ |
|  | Reverse | 5’-TGCATAACTCAACTGTGCAGG-3’ |
| DGCR8 | Forward | 5’-CAAGCAGGAGACATCGGACAAG-3’ |
|  | Reverse | 5’-CACAATGGACATCTTGGGCTTC-3’ |
| Dicer1 | Forward | 5’-TTAACCTTTTGGTGTTTGATGAGTGT-3’ |
|  | Reverse | 5’-AGGACATGATGGACAATT-3’ |
| GAPDH | Forward | 5'-GTCTCCTCTGACTTCAACAGCG-3' |
|  | Reverse | 5'-ACCACCCTGTTGCTGTAGCCAA-3' |
| U6 | Forward | 5’-CTCGCTTCGGCAGCACAT-3’ |
|  | Reverse | 5’-TTTGCGTGTCATCCTTGCG-3’ |

Note: IL-, interleukin-; TNF-α, tumor necrosis factor-α; JAK1, Janus-activated kinase-1; STAT1, signal transducer and activator of transcription 1; miR-155, microRNA-155; DGCR8, DiGeorge syndrome chromosomal region 8; GAPDH, glyceraldehyde-3-phosphate dehydrogenase; RT-qPCR, reverse transcription-quantitative polymerase chain reaction

**Table S2** Primer sequences for RT-qPCR (mouse)

| Gene | Sequence | |
| --- | --- | --- |
| IL-4 | Forward | 5'-TCGGCATTTTGAACGAGGTC-3' |
|  | Reverse | 5'-GAAAAGCCCGAAAGAGTCTC-3' |
| IL-5 | Forward | 5′-TCACCGAGCTCTGTTGACAA-3′ |
|  | Reverse | 5′-CCACACTTCTCTTTTTGGCG-3′ |
| IL-6 | Forward | 5′-TACCACTTCACAAGTCGGAGGC-3′ |
|  | Reverse | 5′-CTGCAAGTGCATCATCGTTGTTC-3′ |
| IL-13 | Forward | 5'-ACGGCAGCATGGTATGGAGTG-3' |
|  | Reverse | 5'-TGGGTCCTGTAGATGGCATTGC-3' |
| TNF-α | Forward | 5′-CCACGCTCTTCTGTCTACTGAACT-3′ |
|  | Reverse | 5′-GGGTCTGGGCCATAGAACTG-3′ |
| JAK1 | Forward | 5’-CTTACCAGGATGCGAATAAA-3’ |
|  | Reverse | 5’-AGTTTCCAAGGTAGCCAGGTAT-3’ |
| STAT1 | Forward | 5’-GATCCCGTACAGATGTCCATGA-3’ |
|  | Reverse | 5’-CCCTCCTGGGCCTGATTA-3’ |
| miR-155 | Forward | 5′-CTGGGTTAATGCTAATTGTGAT-3′ |
|  | Reverse | Reverse Universal Primer |
| Drosha | Forward | 5’-TTATCCATGCACCAGATC-3’ |
|  | Reverse | 5’-TGCATAACTCAACTGTGGAGG-3’ |
| DGCR8 | Forward | 5’-CAAGCAGGAGACCTCTGACAAG-3’ |
|  | Reverse | 5’-TACAATTGACATCTTGGGTTTC-3’ |
| Dicer1 | Forward | 5’-TTAACCTTTTGGTGTTTGATGAGTGT-3’ |
|  | Reverse | 5’-CATCACTGCCACCCAGAAGACTG-3’ |
| GAPDH | Forward | 5'-CATCACTGCCACCCAGAAGACTG-3' |
|  | Reverse | 5'-ATGCCAGTGAGCTTCCCGTTCAG-3' |
| U6 | Forward | 5’-CTCGCTTCGGCAGCACAT-3’ |
|  | Reverse | 5’-TTTGCGTGTCATCCTTGCG-3’ |

Note: IL-, interleukin-; TNF-α, tumor necrosis factor-α; JAK1, Janus-activated kinase-1; STAT1, signal transducer and activator of transcription 1; miR-155, microRNA-155; DGCR8, DiGeorge syndrome chromosomal region 8; GAPDH, glyceraldehyde-3-phosphate dehydrogenase; RT-qPCR, reverse transcription-quantitative polymerase chain reaction
